# Supplementary material for: Antimicrobial Peptides Can Generate Tolerance by Lag and Interfere with Antimicrobial Therapy
Source: Pharmaceutics. 2022 Oct 11;14(10):2169. doi: 10.3390/pharmaceutics14102169 (PMC9611985; doi:10.3390/pharmaceutics14102169)
Supplement: Supplementary file 1 [file pharmaceutics-14-02169-s001.zip › pharmaceutics-1950925 - supplementary.pdf]

pharmaceutics

MDPI

Supplementary Materials

Antimicrobial Peptides Can Generate Tolerance by Lag and Interfere with Antimicrobial Therapy

Daniel Sandín, Javier Valle, Jordi Morata, David Andreu and Marc Torrent

Table S1. MICs in *E. coli* wild type and PolB resistant strain. MIC values are in μM.

Peptide/Strain	*E. coli* wt	PolB
Pleurocidin	0.06	0.06
Polymyxin B	0.01	> 0.2
Dermaseptin	2	2
LL-37	3	3

Table S2. MICs in *E. coli* wild type and evolved. MIC values are in μM.

Antibiotic	*E. coli* wt	PolB	Pleu	LL-37	DMS
Ampicillin	17	17	17	17	17
Kanamycin	1.4	1.4	1.4	1.4	2.8
Ciprofloxacin	42	42	42	42	42
Nalidixic Acid	11	11	11	11	11

Table S3. Summary of relevant mutations in Pleu and LL-37 evolved strains.

Gene	Treatment	Position	Reference Sequence	Mutation	Reference (Sequencing Bioproject)
**yejK-yejL (b2186 -b2187)**	Pleu	2284069	GA	G	--
**araD (b0061)**		66527	C	A	--
		66530	G	GCGACCGAGACC / GCGCCCGAGACCCGCA	--
**gltP (b4077)**		4296267	GCATCCGACATCAACGCCTGATGCGACGCTTAACGCGTCTTATCAGGCCTACGCCAGACAGCGCAATAGCCTGATTTAGCGTGATTTTGTAGGTCGGATAAGGCGTTTA	G	PRJNA476486
		4296272	ATGC	A	--
		4296060	C	T	PRJNA747892
**mlaC (b3192)**		3337787	CG	C	--
**mlaF (b3195)**		3339509	A	C	--
**rhaD (b3902)**		4093986	T	G	--
		4093989	A	ACGCATGCGGATC	--
**sspA (b3229)**		3377363	T	TG	--
**glcE (b4468)**		3125885	A	G	--
**hcr-hcp (b0872-b0873)**		912151	C	T	--
**araD (b0061)**	LL-37	66535	T	A	--
		66540	T	G	--
		66541	C	T	--
**gltP (b4077)**		4296267	GCATCCGACATCAACGCCTGATGCGACGCTTAACGCGTCTTATCAGGCCTACGCCAGA	G	PRJNA476486

	4296272	CAGCGCAATAGCCTGATTTAGCGTGATTTTGTAGGTCGGATAAGGCGTTTAATGC	A	--
	4296078	CCGCGTCTTATCAGGCCTACGCCAGACAGCGCAATAGCCTGATTTAGCGTGATTTTGTAGGTCGGATAAGGCGTTTATGCCGCATCCGACATCAACGCCTGATGCGACGCTTAA	C	PRJNA663118
**valU (b2401)**	2520930	GGGGTGATTAGCTCAGCTGGGAGAGCACCTCCCTTACAAGGAGGGGGTCGGCGGTTCGATCCCGTCATCACCCACCAACTACTTTATGTAGTCTCCGCCGTGTAGCAAGAAATTGAGAAGT	G	PRJNA758726
**mhpC (b0349)**	371761	G	GTAGATTT	
	371760	G	T	
**flhD – insB1 (b1892-b1893)**	1978507	A	AGTCATACTTTTGTTTT	
	1978503	G	T	
	1978504	G	A	
**ychE (b1242)**	1299499	G	A	
	1299500	G	T	
	1299502	A	ATAATCAATTGTTAAATTATT	
**emrY (b2367)**	2481016	T	G	
